# Supplementary material for: Typing of the Gut Microbiota Community in Japanese Subjects
Source: Microorganisms. 2022 Mar 20;10(3):664. doi: 10.3390/microorganisms10030664 (PMC8954045; doi:10.3390/microorganisms10030664)
Supplement: Supplementary file 1 [file microorganisms-10-00664-s001.zip › 20220227_Supplementary_Figure_Legends.pdf]

Supplementary Figure S1. Model fitting for partitioning around medoids (PAM) and Dirichlet multinominal mixtures (DMM) models. The optimal number of gut microbiota communities were identified based on average silhouette width for PAM (a) and Laplace approximation to the negative log model evidence of the DMM model (b). The greatest average silhouette width and minimum Laplace approximation are indicated by the dotted line

Supplementary Figure S2. Abundance of bacterial groups relating to the family *Ruminococcaceae* in five PAM-identified community types. Box plots showing the abundance of the genus *Ruminococcus* belonging to family *Ruminococcaceae* (hatched), Unclassified genera belonging to the family *Ruminococcaceae* (horizontal line) and the family *Ruminococcaceae* (filled)

Supplementary Figure S3. Odds ratios for various diseases in each of the PAM-identified community types based on a more detailed disease background. The odds ratio for various diseases in each PAM-identified community type was calculated based on the number of individuals with each of the indicated diseases. Statistically significant differences ($p < 0.05$) were calculated based on the Wald test. Red boxes indicate significantly higher odds ratios of the diseases in comparison to type D. Gray boxes indicate no statistically significant difference with type D

Supplementary Figure S4. Taxonomic composition of the microbial community at the genus level for 15 DMM model-identified community types. Cumulative bar charts for the abundance of frequently detected genera (a) and a heatmap of the mean abundance values of frequently detected genera (b). The frequently detected genera were comprised of thirty-six genera, which were detected in more than 50% of the subjects

Supplementary Figure S5. Odds ratios of various diseases in each of the 15 DMM model-identified community types. The odds ratio for various diseases in each DMM model-identified type was calculated based on the number of individuals with each of the indicated diseases. Statistically significant differences ($p < 0.05$) were calculated based on the Wald test. Red boxes indicate significantly higher odds ratios for the diseases in comparison to type 10. Gray boxes indicate no significant difference with type 10

Supplementary Figure S6. The α-diversity and principal coordinate analysis (PCoA) plots of gut microbiota for 15 community types stratified based on the Dirichlet multinominal mixture (DMM) model analysis. The α-diversity assessed by Chao 1 index (ASV richness estimation) (a) and Shannon index (ASV evenness estimation) (b). Statistically significant differences in α-diversity indices among the groups were

evaluated using one-way ANOVA with Benjamini-Hochberg correction. Statistical significance ($p < 0.05$) is indicated by the different letters (a and b). β-diversity represented by principal coordinate analysis plots based on Bray-Curtis dissimilarity; axis 1 and axis 2 (c) and axis 3 and axis 4 (d). Statistically significant differences in β-diversity among the groups were confirmed using PERMANOVA ($p = 0.001$)
