# Supplementary material for: Typing of the Gut Microbiota Community in Japanese Subjects
Source: Microorganisms. 2022 Mar 20;10(3):664. doi: 10.3390/microorganisms10030664 (PMC8954045; doi:10.3390/microorganisms10030664)
Supplement: Supplementary file 1 [file microorganisms-10-00664-s001.zip › S20211231Microbiota_Profile_supplFig_ver11.pptx]

## Slide 1
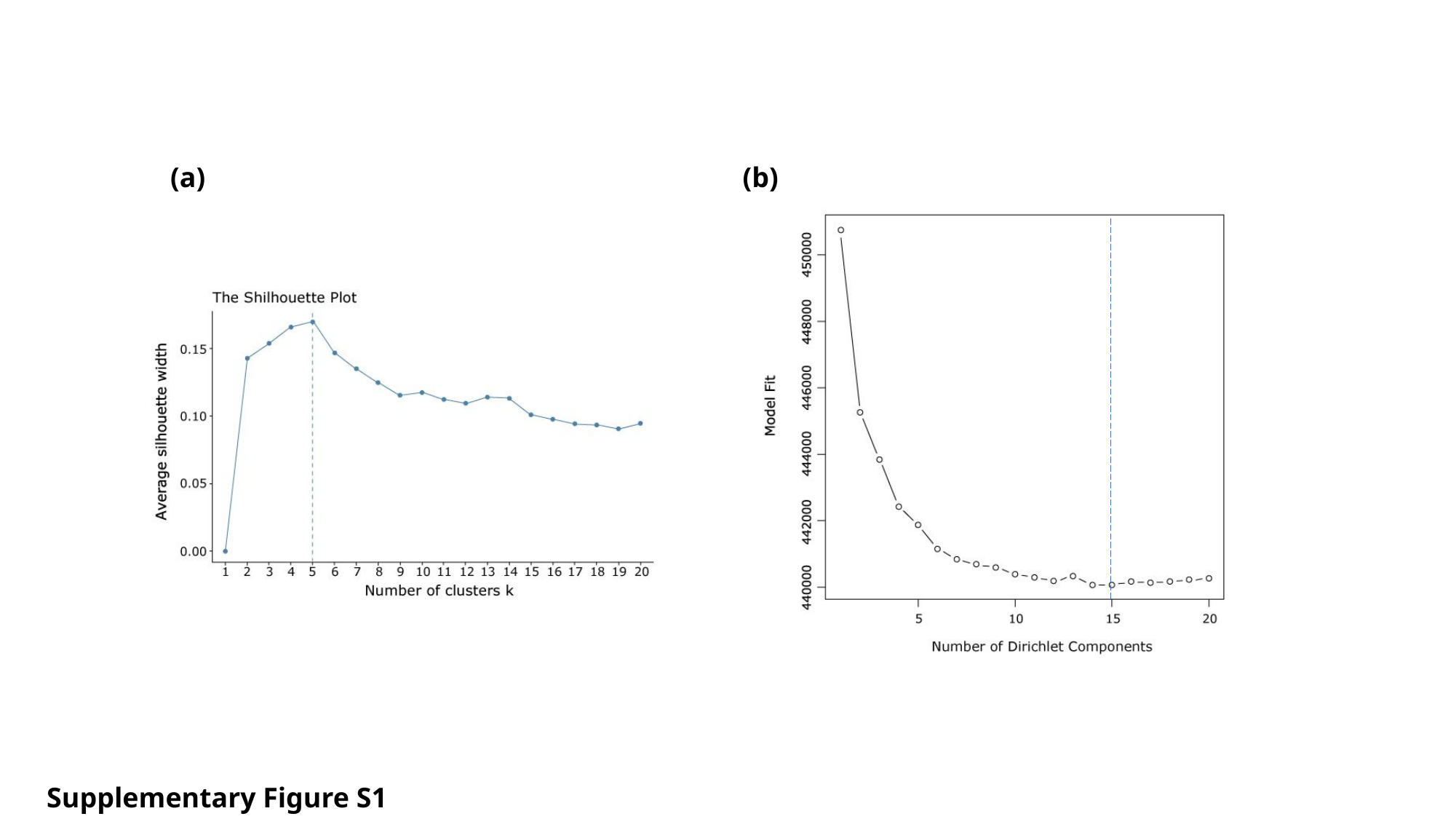

(a)
(b)
Supplementary Figure S1

## Slide 2
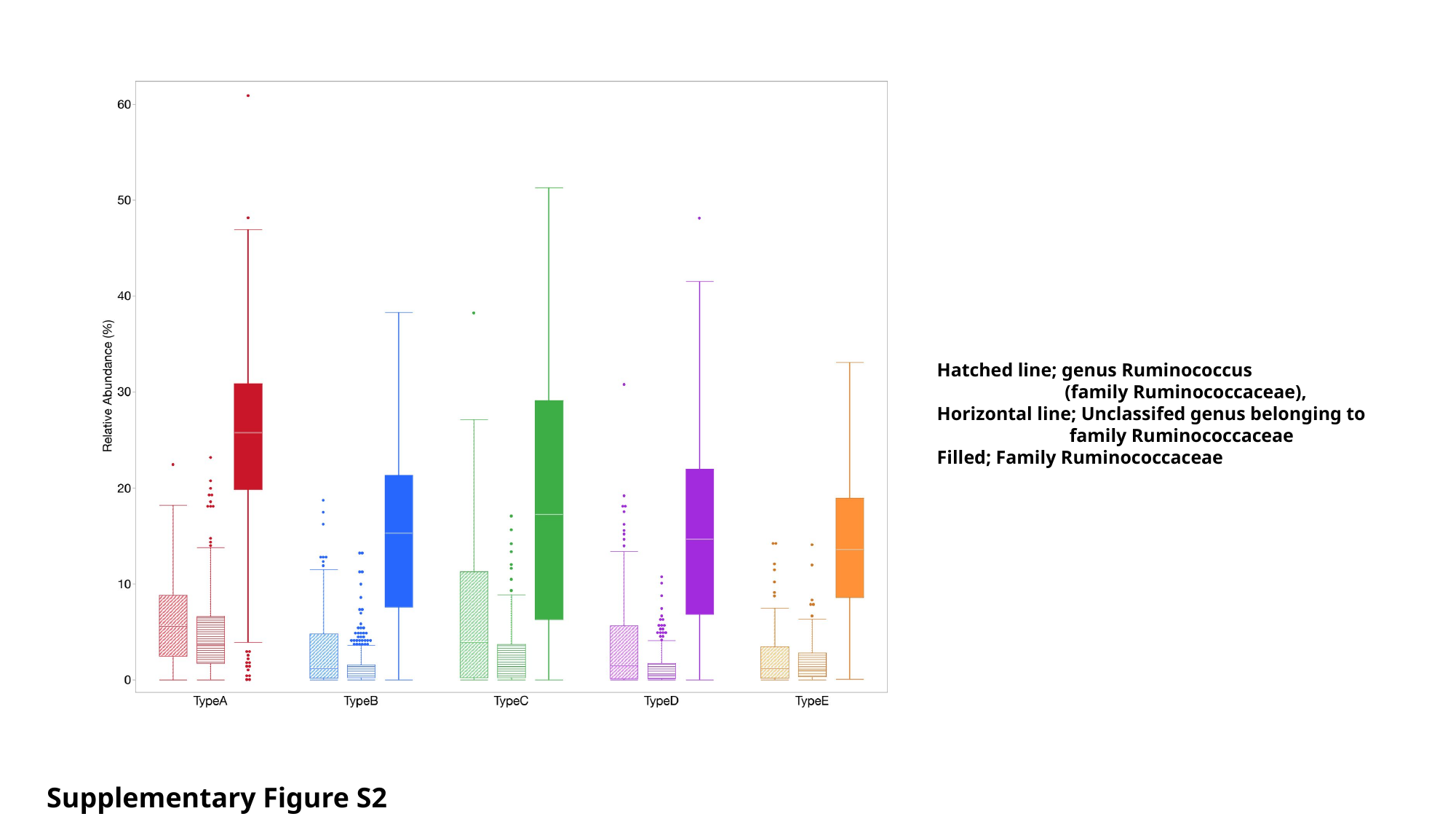

Hatched line; genus Ruminococcus
 (family Ruminococcaceae),
Horizontal line; Unclassifed genus belonging to
 family Ruminococcaceae
Filled; Family Ruminococcaceae
Supplementary Figure S2

## Slide 3
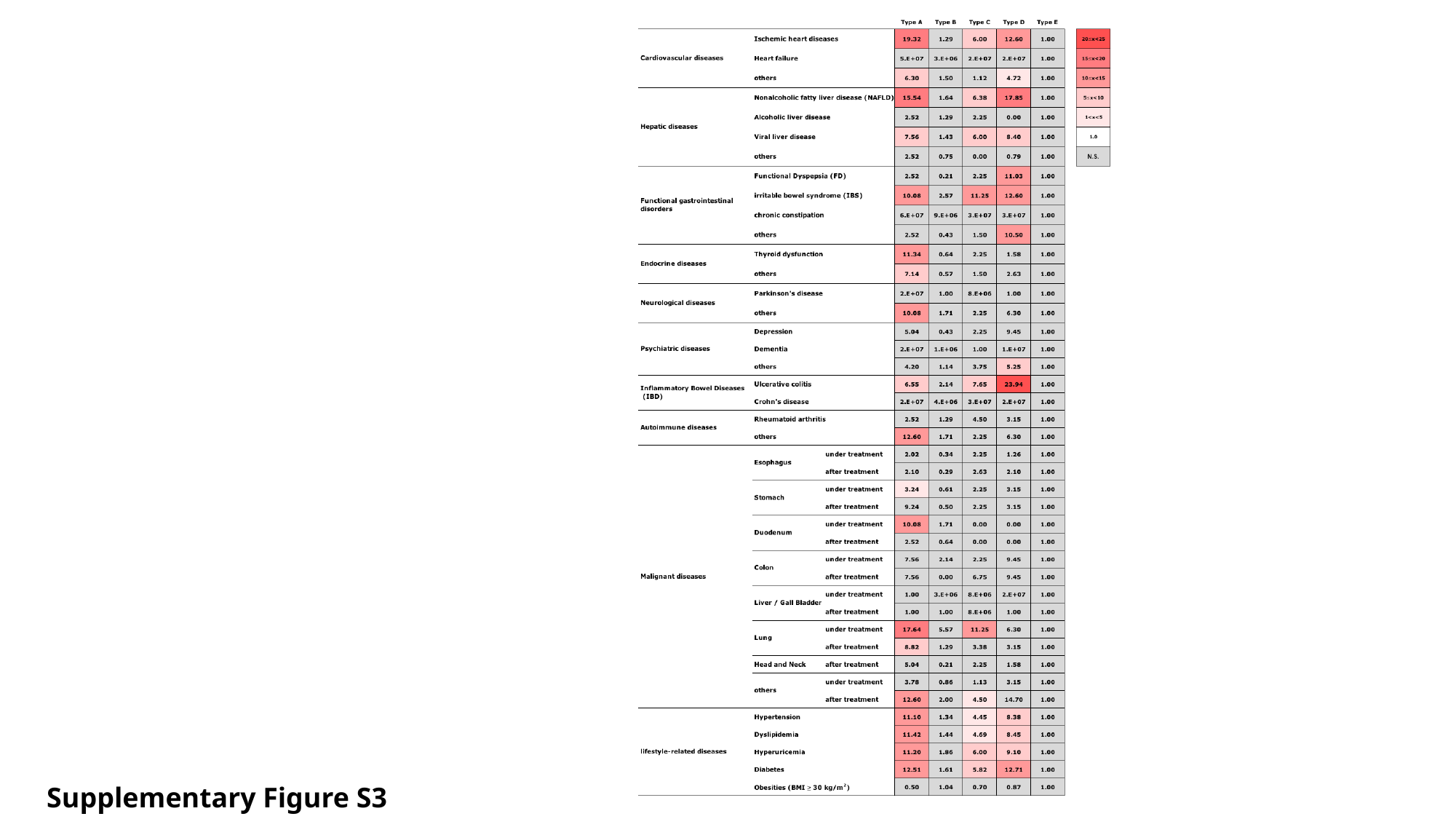

Supplementary Figure S3

## Slide 4
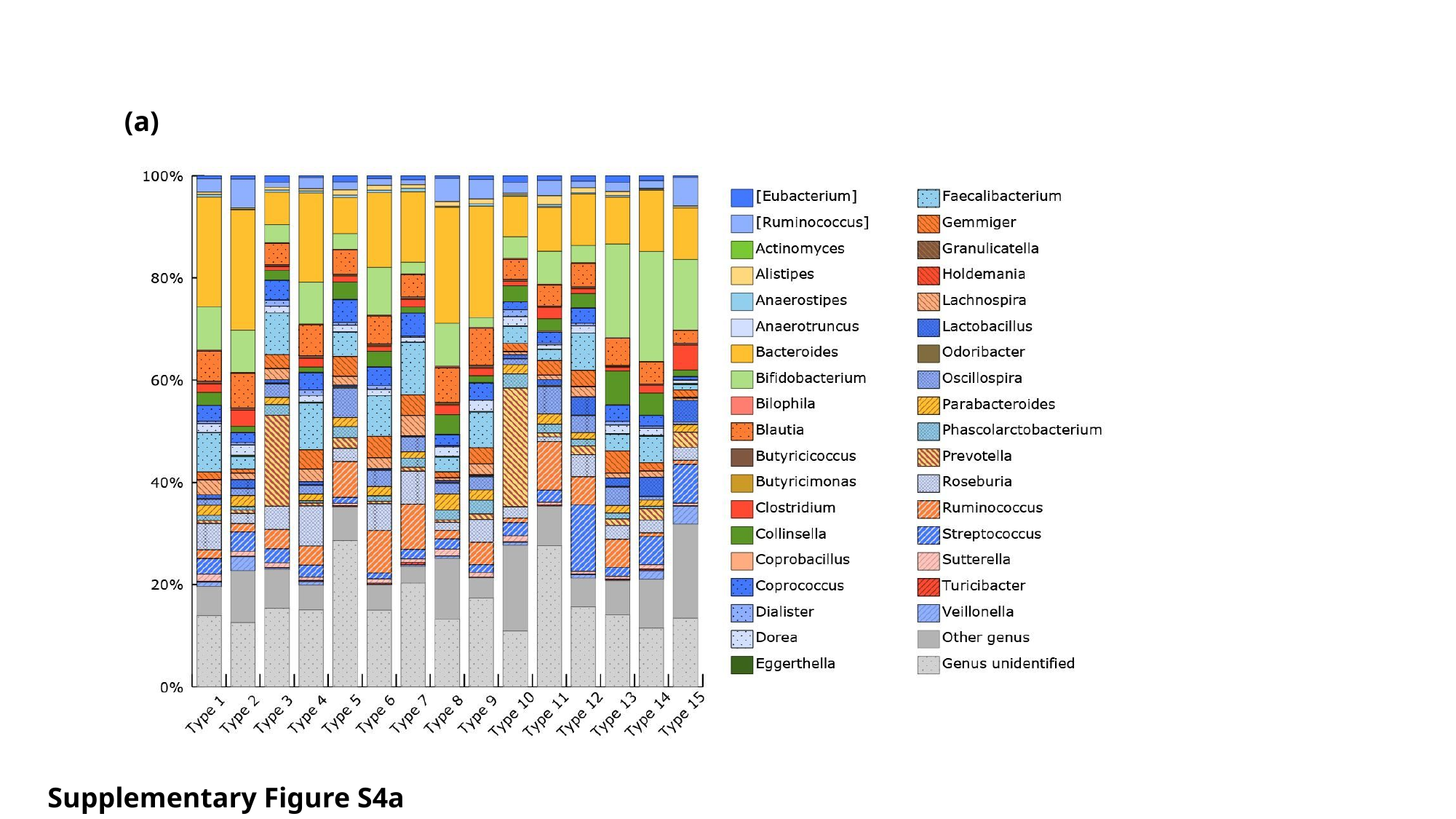

(a)
Supplementary Figure S4a

## Slide 5
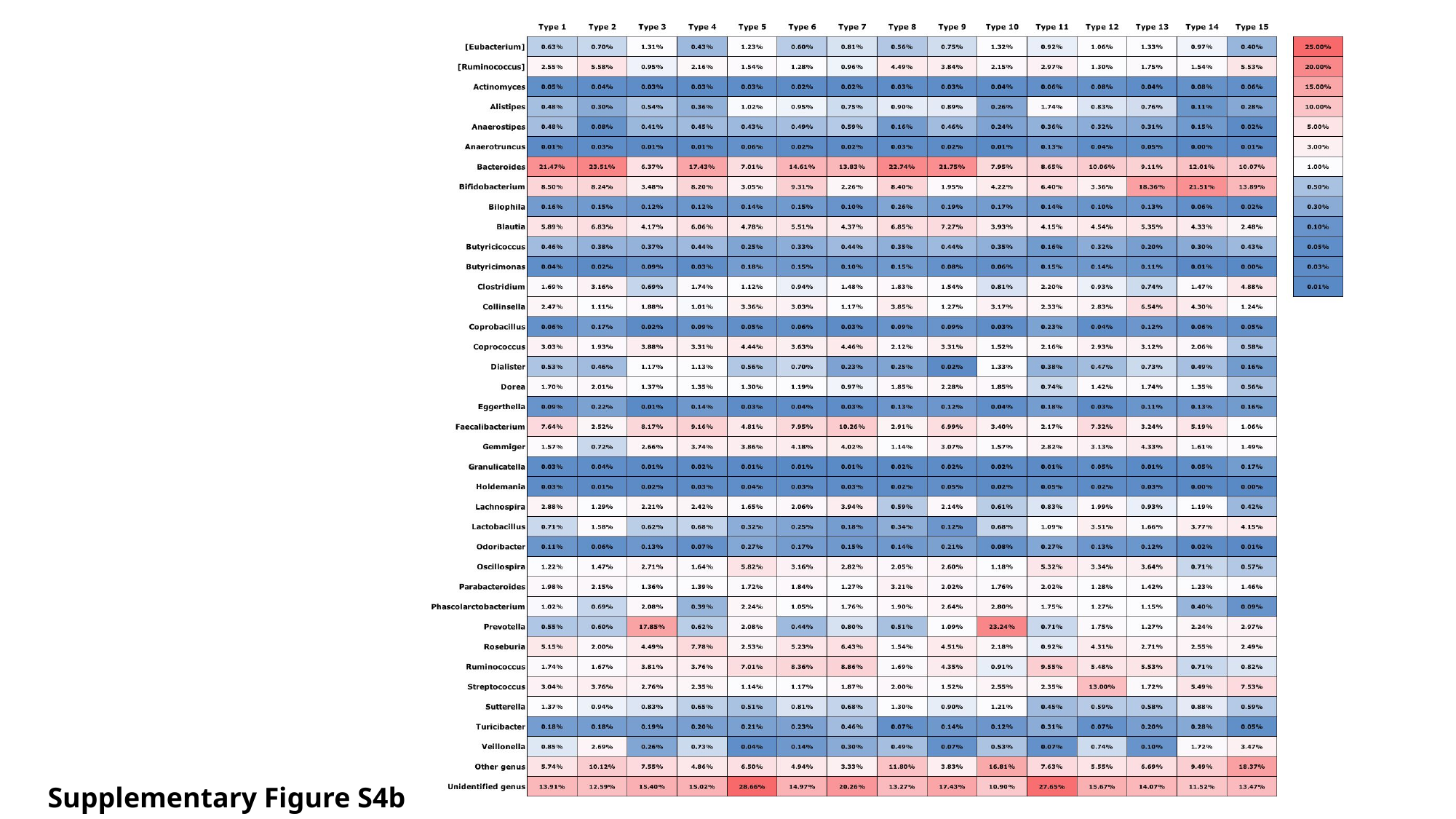

Supplementary Figure S4b

## Slide 6
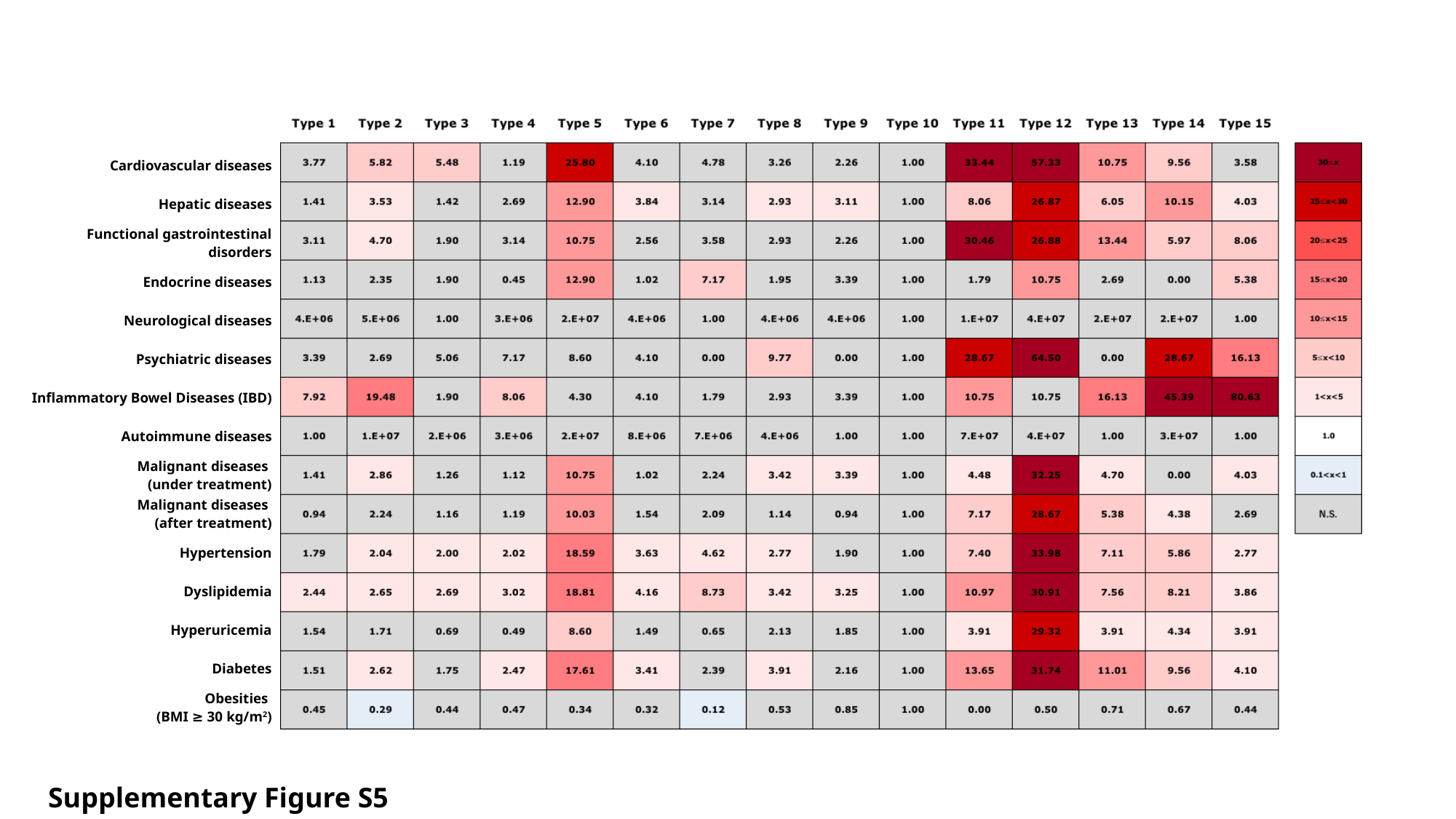

| Cardiovascular diseases |
| --- |
| Hepatic diseases |
| Functional gastrointestinal disorders |
| Endocrine diseases |
| Neurological diseases |
| Psychiatric diseases |
| Inflammatory Bowel Diseases (IBD) |
| Autoimmune diseases |
| Malignant diseases (under treatment) |
| Malignant diseases (after treatment) |
| Hypertension |
| Dyslipidemia |
| Hyperuricemia |
| Diabetes |
| Obesities (BMI ≥ 30 kg/m2) |
Supplementary Figure S5

## Slide 7
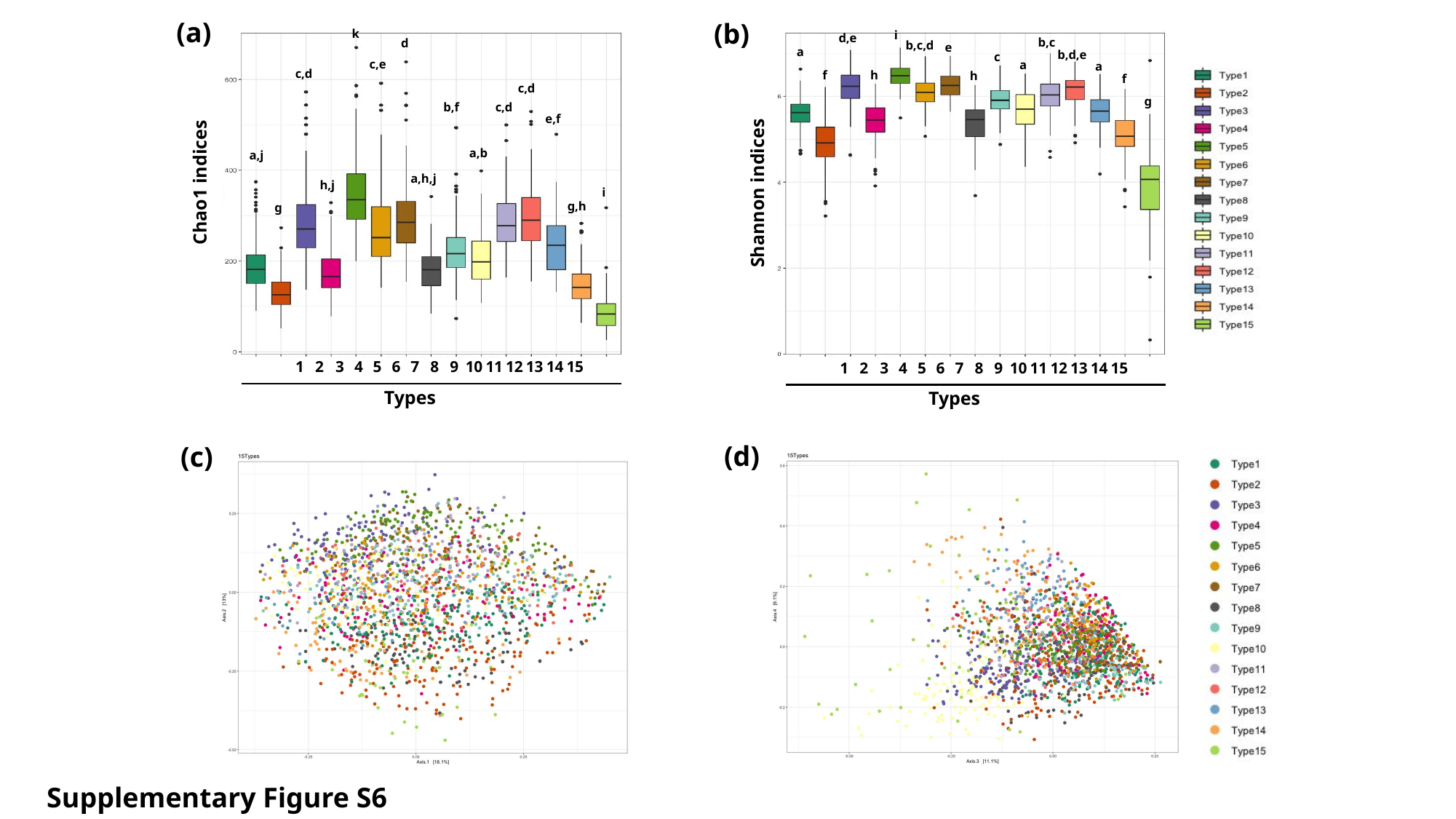

(a)
(b)
k
i
d,e
b,c
d
b,c,d
e
a
b,d,e
c
c,e
a
a
c,d
h
f
h
f
c,d
g
c,d
b,f
e,f
a,b
a,j
Chao1 indices
a,h,j
h,j
 Shannon indices
i
g,h
g
 1 2 3 4 5 6 7 8 9 10 11 12 13 14 15
 1 2 3 4 5 6 7 8 9 10 11 12 13 14 15
Types
Types
(d)
(c)
Supplementary Figure S6
